# Eccentric exercise‐induced muscle damage and inflammation in conjunction with high‐altitude decompression in adults

**DOI:** 10.14814/phy2.70147

**Published:** 2024-12-12

**Authors:** Frode Gottschalk, Mikael Gennser, Mattias Günther, Ola Eiken, Antonis Elia

**Affiliations:** ^1^ Division of Environmental Physiology Swedish Aerospace Physiology Center, KTH Royal Institute of Technology Stockholm Sweden; ^2^ Department of Neuroscience, Experimental Traumatology KI Karolinska Institutet Stockholm Sweden; ^3^ Department of Physiology and Pharmacology KI Karolinska Institutet Stockholm Sweden

**Keywords:** aviation, biomarkers, decompression, eccentric exercise, high‐altitude, venous gas emboli

## Abstract

This study aimed to investigate the effect of eccentric exercise on exercise‐induced muscle damage (EIMD) and inflammation on high‐altitude‐induced venous gas emboli (VGE). Subjects were exposed to an altitude of 24,000 ft. for 90 min, with either prior eccentric exercise (ECC) or no exercise (Control) 24 h before. Blood samples were collected at baseline (T_0_), before (T_1_), and after (T_2_) altitude exposures. VGE load was evaluated using the Eftedal‐Brubakk (ΕΒ) scale. Creatine kinase (CK) and myoglobin were used to assess muscle damage, while interleukin‐6 (IL‐6), tumor necrosis factor alpha (TNF‐α), C‐reactive protein (CRP), and fibrinogen were used to evaluate inflammation. ECC showed higher EB‐scores during altitude exposures [median(range), 3(0–5)] than Control [1(0–4), *p* = 0.019]. Increases in myoglobin (+35%, *p* = 0.012), CK (+130%, *p* < 0.001), IL‐6 (+72%, *p* = 0.02), and CRP (+63%, *p* = 0.004) were observed from T_0_ to T_1_ in ECC, but not Control. Significantly higher levels of myoglobin (*p* = 0.033), CK (*p* < 0.001), IL‐6 (*p* = 0.016), and CRP (*p* = 0.002) were noted in the ECC compared to Control at T_1_. IL‐6 increased from T_1_ to T_2_ in ECC (*p* = 0.005), with higher levels than Control at T_2_ (*p* = 0.046). A correlation was found between EB‐scores and T_1_ myoglobin levels (*r*_*s*_ = 0.450; *p* = 0.004), and to T_1_‐T_2_ IL‐6 changes (*r*_*s*_ = 0.396; *p* = 0.037). Eccentric EIMD followed by inflammation is associated with a higher decompression strain, with VGE load aggravating systemic inflammation.

## 1 INTRODUCTION

Exposure to reduced atmospheric pressure results in the liberation of dissolved gas from saturated tissues and can lead to the formation of bubbles within tissue and blood (Stepanek, 2002). It is generally accepted that these bubbles are the primary cause of decompression sickness (DCS) (Nishi et al., 2003), and a large number of observable bubbles in the venous circulation (VGE) is associated with an increased risk of DCS (Eftedal et al., 2007; Eiken et al., 2023; Sawatzky, 1991). Thus, the presence of VGE in the right ventricle of the heart is commonly used as a measure of decompression strain. Gas saturation alone is insufficient to explain the production of VGE observed during decompression, as de novo bubble formation would require a supersaturation of 10.0 MPa (Jones et al., 1999). Hitherto, the main hypothesis is that bubbles originate from small (1–3 μm in diameter) precursor micronuclei or even smaller volumes of free gas (Arieli & Marmur, 2011) found in tissue and blood (Arieli & Marmur, 2011; Blatteau et al., 2006; Vann et al., 1980).

Evidence suggests that exercise dominated by eccentric contractions causes more VGE to form during subsequent decompression (Gottschalk et al., 2023; Gottschalk, Gennser, Günther, et al., 2024). The increased number of VGE may increase the risk of decompression sickness (Damato et al., 1963; Hughes & Eckenhoff, 1986; Nishi et al., 1982; Vann, 1982; Vann & Gerth, 1995). Although the precise mechanism(s) underlying such response remain unknown, it is likely that eccentric exercise increase the presence of micronuclei. Eccentric contractions result in exercise‐induced muscle damage (EIMD) with structural damage to tissue and subsequent efflux of myocellular proteins (Lindstedt et al., 2001). The damaged tissue initiates an inflammatory process with local vascular hyperpermeability (Hotta et al., 2018) and systemic elevation in biomarkers indicative of inflammation (Peake et al., 2017). Thus, the observed increase in VGE following eccentric exercise could be due to (i) generation of new micronuclei due to cavitation phenomena resulting from strong muscular contractions (Harvey, 1945; Unsworth et al., 1971; Whitaker et al., 1945), (ii) local hyperpermeability making it easier for micronuclei to transverse the endothelium wall (Hotta et al., 2018), or (iii) an alteration in the surface tension of pre‐existing micronuclei, potentially prolonging their lifespan (Philp et al., 1972; Thorsen et al., 1989; Van Liew & Raychaudhuri, 1997).

While previous studies suggest that eccentric exercise increases VGE load, it is important to note that markers indicative of inflammation has not previously been assessed (Gottschalk et al., 2023; Gottschalk, Gennser, Günther, et al., 2024). Exposure to decompression has been associated with an inflammatory response. While several studies have investigated these changes following hyperbaric decompression (Connolly et al., 2023; Ersson et al., 2002; Gempp et al., 2012; Nyquist et al., 2007; Rocco et al., 2021; Spisni et al., 2017; Žarak et al., 2021), less is known about the effects of hypobaric decompression on inflammatory responses. It is possible that an already activated immune system caused by eccentric exercise may be further stimulated by decompression exposure, perhaps leading to greater decompression strain.

Accordingly, this study aimed to investigate the effect of eccentric exercise on muscle damage and inflammation and explore their potential role on high‐altitude‐induced VGE.

## 2 METHOD

### 2.1 Ethics approval

This study received ethics approval from the Swedish National Ethics Review (reference number 2023–06714‐02), and all experimental procedures conformed to the *Declaration of Helsinki*, except for registration in a database.

### 2.2 Subjects

Twenty‐one healthy, non‐smoking adults (16 men; 5 women) volunteered to participate in the study. Their age, weight, and body mass index [mean (range)] were 32 (21–57) yrs., 82 (68–110) kg, and 25 (21–31) kg/m^2^, respectively. Prior to the first hypobaric exposure, subjects underwent a physical examination of the ear drums, heart, lungs, and gross neurological functions. Subjects with a medical history of cardiorespiratory or metabolic disorders, chronic infections, disorders that necessitated the use of medication, claustrophobia, or difficulties equalizing pressure in the middle ears were excluded from the study. Detailed information about the experimental procedures, associated risks, and potential benefits was provided to the subjects, both in written and oral form, prior to obtaining their informed written consent.

### 2.3 Experimental overview

This study is part of a larger project examining the effects of eccentric exercise on high‐altitude‐induced VGE formation in humans. The blood samples analyzed herein were collected in connection with two previous investigations (Gottschalk et al., 2023; Gottschalk, Gennser, Günther, et al., 2024). In the second study (Gottschalk, Gennser, Günther, et al., 2024) the subjects conducted two different eccentric exercise interventions (upper body with or without lower body exercise) and one control test each (no eccentric exercise). Consequently, there is unequal number of subjects between the intervention and control groups (Table 1). All experimental procedures were conducted at the Division of Environmental Physiology of the Royal Institute of Technology in Solna, Sweden.

**TABLE 1:** Number of subjects in each condition and group, along with the median maximum EB scores during altitude exposures.

	Eccentric exercise	Control	VGE_visible_	VGE_no‐visible_
Total number	31	21	35	17
Eccentric exercise	31	0	25	6
Control	0	21	10	11
Median EB‐score	3^a^	1	3	0

Abbreviations: EB‐score, Eftedal‐Brubakk score; VGE, venous gas emboli.

^a^ Significance between ECC and Control (p=0.019). Mann–Whitney *U*‐test; *n* = 52.

### 2.4 Experimental protocol

During each experimental visit, subjects reported to the laboratory after abstaining from caffeine‐ and alcohol‐containing beverages for 12 and 24 h, respectively. Furthermore, before and throughout each experimental session, individuals were told to refrain from tobacco use for 12 h, strenuous physical activity for 72 h, and diving or flying for 7 days. Blood samples were collected prior to the exercise/control intervention (T_0_), as well as before (T_1_) and immediately after (T_2_) the altitude exposures.

### 2.5 Eccentric exercise

Before the eccentric exercise, subjects underwent a 5‐min seated rest period after which 4 mL whole blood samples were drawn from the antecubital vein of the subject's arm (T_0_). The eccentric exercise protocols are described in detail elsewhere (Gottschalk, Gennser, Eiken, & Elia, 2024). Briefly, the eccentric upper‐body exercise protocol involved 15 repetitions of 1‐min eccentric work bout, interspersed with 1‐min rest periods, performed on a custom‐made arm ergometer (Elmer et al., 2013; Gottschalk, Gennser, Eiken, & Elia, 2024). The subjects were instructed to resist the pedals while maintaining a cadence of 50 rpm with a power output corresponding to 60% of the force during maximal voluntary contraction of the elbow flexors. The lower‐body eccentric exercise protocol compromised ten sets of ten weighted squats, with a 2‐min recovery interval between sets. The squat bar was loaded with weights corresponding to 60% of the force generated during knee extension maximal voluntary contractions (MVC). To emphasize eccentric contractions, the subjects were instructed that the downward phase of the squat would last for 2–3 s, whereas the experimenter would assist during the upward phases. The experimenter aided during the upward phase if necessary to complete the targeted 100 squats. Throughout the exercises, subjects were verbally encouraged to exert maximal effort.

### 2.6 Preliminary measures

Subjects reported to the laboratory dressed in sports clothes (i.e., shorts, short‐sleeved t‐shirt, and sneakers) 24‐h after: (i) completing the eccentric exercise protocol (ECC) or (ii) avoidance of exercise (Control). Thereafter, they completed a 5‐min seated rest period after which two 4 mL whole blood samples were drawn (T_1_). Thereafter, the subjects performed 50 deep, unloaded knee squats over a 5‐min period in an attempt to equalize the subjects' exercise state prior to the altitude exposure.

### 2.7 Hypobaric exposure

Immediately after the 50 knee squats, the subjects entered the hypobaric chamber and were placed on a gurney on their left side. Just before (30–60 s) the start of the pressure reduction and for the entirety of the hypobaric exposure, the subjects wore a full‐face breathing mask (Poseidon Diving Systems AB, Göteborg) and breathed 100% oxygen. During each simulated altitude exposure, subjects were accompanied by an inside experimenter who conducted the VGE measurements. The inside experimenter breathed 100% O_2_ through a full‐face diving mask and a demand valve during, and for 1 h preceding, each experiment.

Subjects were exposed to a pressure corresponding to 24,000 ft. altitude (7315 m above sea level ≈40 kPa) continuously for 90 min. This protocol has been shown to successfully provoke the formation of VGE (Ånell et al., 2019, 2022; Elia et al., 2021; Gottschalk et al., 2023). The pressure in the chamber was lowered and raised at a rate of 5000 ft. • min^−1^ (1524 m • min^−1^). During the 90‐min exposure, the participant's heart rate and cardiac output was recorded (Physioflow PF07, Enduro, Manatec Biomedical, Paris, France), as well as the SpO_2_ levels (Radical 7 Monitor MASIMO SET, USA). The temperature within the hypobaric chamber was 21 ± 1°C throughout the 24,000 ft. exposure. After the 90‐min altitude exposure was completed and upon reaching sea level (≈5 min), two additional 4 mL whole blood samples were collected (T_2_).

### 2.8 VGE and DCS assessment

During the altitude exposures, all ultrasonographic scans and VGE evaluations were conducted by the same sonographer (Elia et al., 2022), in accordance with the guidelines published by (Møllerløkken et al., 2016). The presence of VGE was detected from four‐chamber images using an ultrasound imaging system (Philips Ultrasound, CX50, Bothell, WA, USA), equipped with a 1–5 MHz phased‐array transducer (S5‐1). The incidence of VGE was assessed while the subjects remained lying on their left side on the gurney, at rest (5 min intervals) and after three unloaded knee‐ and elbow‐flex provocations (15 min intervals). During a period of ten cardiac cycles, cardiac images were acquired, and the prevalence of VGE was assessed using the Eftedal‐Brubakk (EB) 6‐graded scale: (0–5): 0 = no visible bubbles; 1 = occasional bubbles; 2 = at least one bubble every fourth heartbeat; 3 = at least one bubble every heartbeat; 4 = at least one bubble/cm^2^; 5 = whiteout single bubbles cannot be discriminated. End‐point criteria for any altitude exposure included a single VGE score of 4 or 5, arterial bubbles, and/or symptoms indicative of DCS. Evaluation of VGE was conducted by the experimenter inside the chamber (on‐line), and subsequently, the images were further assessed after the exposure (offline). The researcher responsible for rating the VGE scores off‐line was blinded to the protocols, and the offline evaluation was used for statistical analysis. The Kisman Integrated Severity Score (KISS) was calculated for each subject according to the following formula:

$$\text{KISS}=\frac{100}{4^{\alpha }\left(t_{n}-t_{1}\right)}\sum_{i=1}^{n}\frac{\left(t_{i+1}-t_{i}\right)\left(d_{i+1}^{\alpha }+d_{i}^{\alpha }\right)}{2}$$

where *t*_*i*_ is time of observation in minutes after reaching altitude (for time points 1 to n), *d*_*i*_ ultrasound score (grades 0–5) observed at time *t*_*i*_ and *α* = 3 (the parameter α takes into account that the bubble grade is not a linear measure of bubble quantity) (Kisman et al., 1978). The total KISS for each subject were calculated based on peak EB score every 5 min, regardless of sedentary, knee‐ or elbow‐flex provocations.

### 2.9 Biomarkers

Myoglobin and creatine kinase (CK) were used to assess the degree of muscle damage, and tumor necrosis factor‐alpha (TNF‐α), interleukin‐6 (IL‐6), interleukin‐1β (IL‐1β), fibrinogen, and C‐reactive protein (CRP) were used to evaluate the level of inflammation.

### 2.10 Analysis of biomarkers

After collection, plasma tubes (Becton, Dickinson and Company, Franklin Lakes, USA, ref. 367,862) were gently inverted and centrifuged (Nüve, Kabul, Turkey) for 10 min in 20°C at 2000 ***g*** (i.e., for myoglobin and fibrinogen) or for 15 min in 20°C at 1000 ***g*** (i.e., for CK, TNF‐α, IL‐6, IL‐1β/IL‐1F2, and CRP). Plasma samples were then aliquoted in Eppendorf tubes and were stored at −80°C until subsequent analysis. Samples were prepared and analyzed according to the manufacturer's instructions with CK quantified using an activity assay (CK, Sigma‐Aldrich, MAK116, CV ~ 2%) and myoglobin, TNF‐α, IL‐6, 1β/IL‐1F2, CRP, and fibrinogen being assessed using an enzyme‐linked immunosorbent assay technique (myoglobin, Abcam, ab171580, CV ~ 3%; TNF‐α, R&D Systems, HSTA00E, CV ~ 4%; IL‐6, R&D Systems, HS600C, CV ~ 3%; IL‐1β/IL‐1F2, R&D Systems, HSLB00D, CV ~ %; CRP, R&D Systems, DCRP00B, CV ~ 3%; fibrinogen, Abcam, ab241383, CV ~ 4%). Samples were washed on a microplate washer (ThermoFisher Scientific, Vantaa, Finland) and read on a spectrophotometer (ThermoFisher Scientific, Joo Koon, Singapore).

### 2.11 Statistical analysis

All data were analyzed using IBM SPSS Statistics software version 28 (IBM Corp., Armonk, NY, USA). The Shapiro–Wilk test was used to assess the data distribution, and only non‐parametric tests were used for statistical inference. Friedman's test and Wilcoxon signed‐rank test were used for paired groups comparisons, while the Mann–Whitney *U*‐test was used for non‐paired groups comparisons. Correlation between maximum VGE score (VGE_max_) and/or KISS and plasma levels of CK, myoglobin, IL‐6, TNF‐α, CRP and fibrinogen were tested using Spearman's correlation test. The effect size was evaluated using Wilcoxon effect size (r). Differences in plasma levels in paired and unpaired groups were presented as absolute changes. Unless otherwise stated, data are reported as means ± SD and significance was accepted at *p* < 0.05.

## 3 RESULTS

### 3.1 VGE

Blood samples were successfully collected before and after 52 simulated altitude exposures (ECC 31; Control 21). EB‐scores were higher in the ECC [median (range), 3 (0–5)] compared with Control [1 (0–4), *p* = 0.019]. Similarly, KISS was higher in the ECC protocol [9.7 *±* 9.4 arbitrary units (a.u.)] compared to Control (4.4 *±* 6.7 a.u., *p* = 0.046). Furthermore, exposures with visible bubbles and those without were split into two groups (VGE_visible_; VGE_no‐visible_) in order to compare the effect of circulating VGE (Table 1).

### 3.2 Biomarkers—ECC and control

**FIGURE 1:**
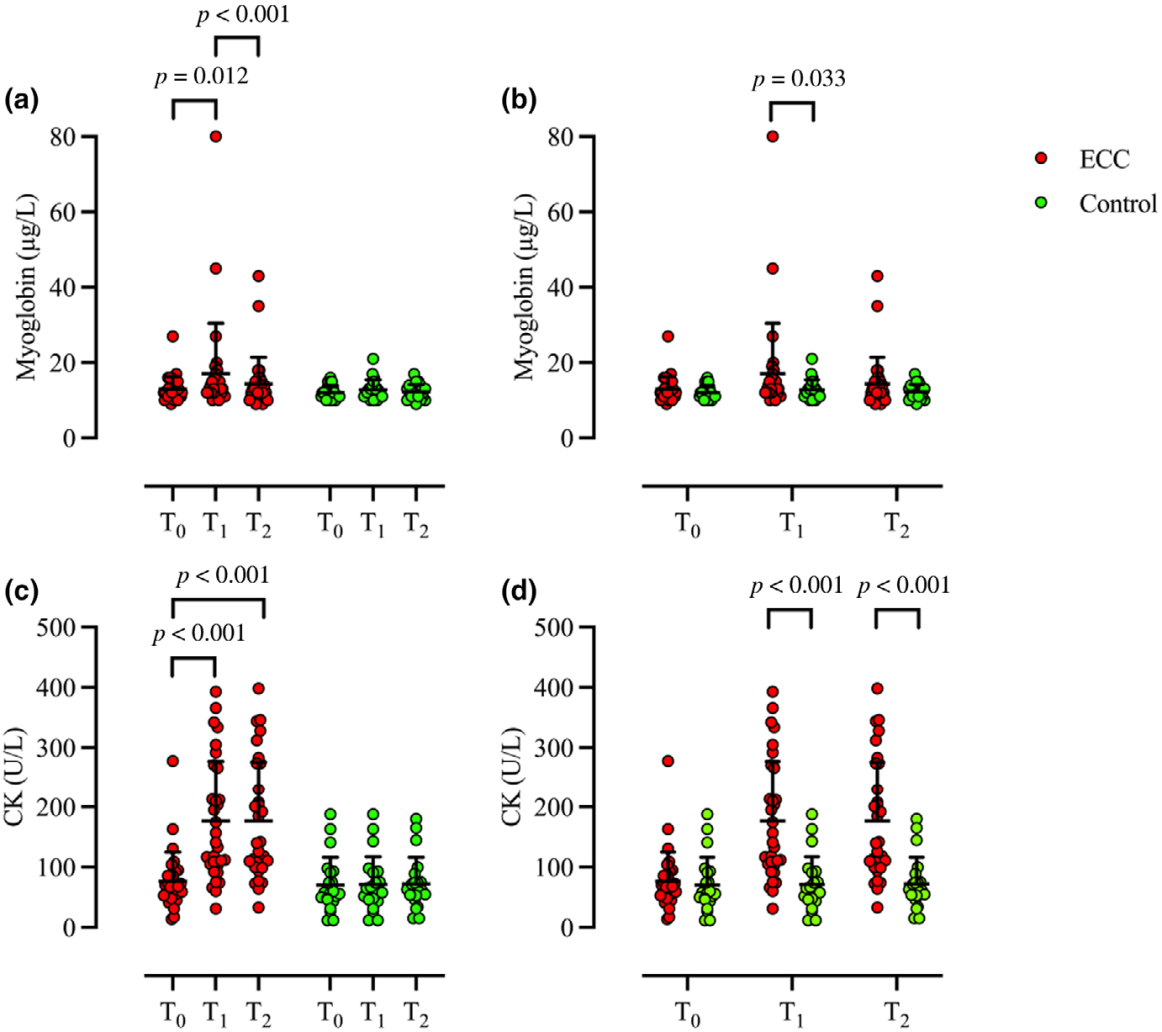
Individual (circle) and mean ± SD (black) concentrations of myoglobin (a and b) and CK (c and d) comparison at timepoints T_0_, T_1_, and T_2_ within (a and c) and between (b and d) ECC (red) and Control (green). Wilcoxon signed‐rank test [within (a and c)] and Mann–Whitney *U*‐test [between (b and d)]; ECC, *n* = 31; Control, *n* = 21. CK, creatine kinase; ECC, eccentric exercise.

Significant differences in myoglobin levels were noted within ECC (*p* = 0.001), with elevated levels from T_0_ (12.9 ± 3.2 μg/L) to T_1_ (17.3 ± 13.3 μg/L, *p* = 0.012, *r* = 0.320) and reduced levels between T_1_ and T_2_ (14.3 ± 7.1 μg/L, *p* < 0.001, *r* = 0.447), while no differences were observed between T_0_ and T_2_ (*p* = 0.478). There was no difference in myoglobin levels between any timepoints (T_0_ 12.0 ± 7.1 μg/L; T_1_ 12.6 ± 1.9 μg/L; T_2_ 12.2 ± 2.0 μg/) in the control condition (*p* = 0.712). No difference in myoglobin levels were observed between ECC and control at T_0_ (*p* = 0.318) or T_2_ (*p* = 0.553) but were higher at T_1_ in ECC compared to control (*p* = 0.033, *r* = 0.293) (Figure 1). Significant changes in CK levels were observed within ECC (*p* < 0.001), with higher levels at T_1_ (176 ± 100 U/L, *p* < 0.001, *r* = 0.558) and T_2_ (177 ± 99 U/L, *p* < 0.001, *r* = 0.542), compared to T_0_ (76 ± 48 U/L) while no difference was seen between T_1_ and T_2_ (*p* = 0.604). No difference was observed in CK levels within the control condition (T_0_ 70 ± 46 U/L, T_1_ 72 ± 42 U/L, T_2_ 73 ± 43 U/L, *p* = 0.273). There was no difference in CK between ECC and Control at T_0_ (*p* = 0.396), but higher levels were noted in ECC at T_1_ (*p* < 0.001, *r* = 0.606) and T_2_ (*p* < 0.001, *r* = 0.619) (Figure 1).

**FIGURE 2:**
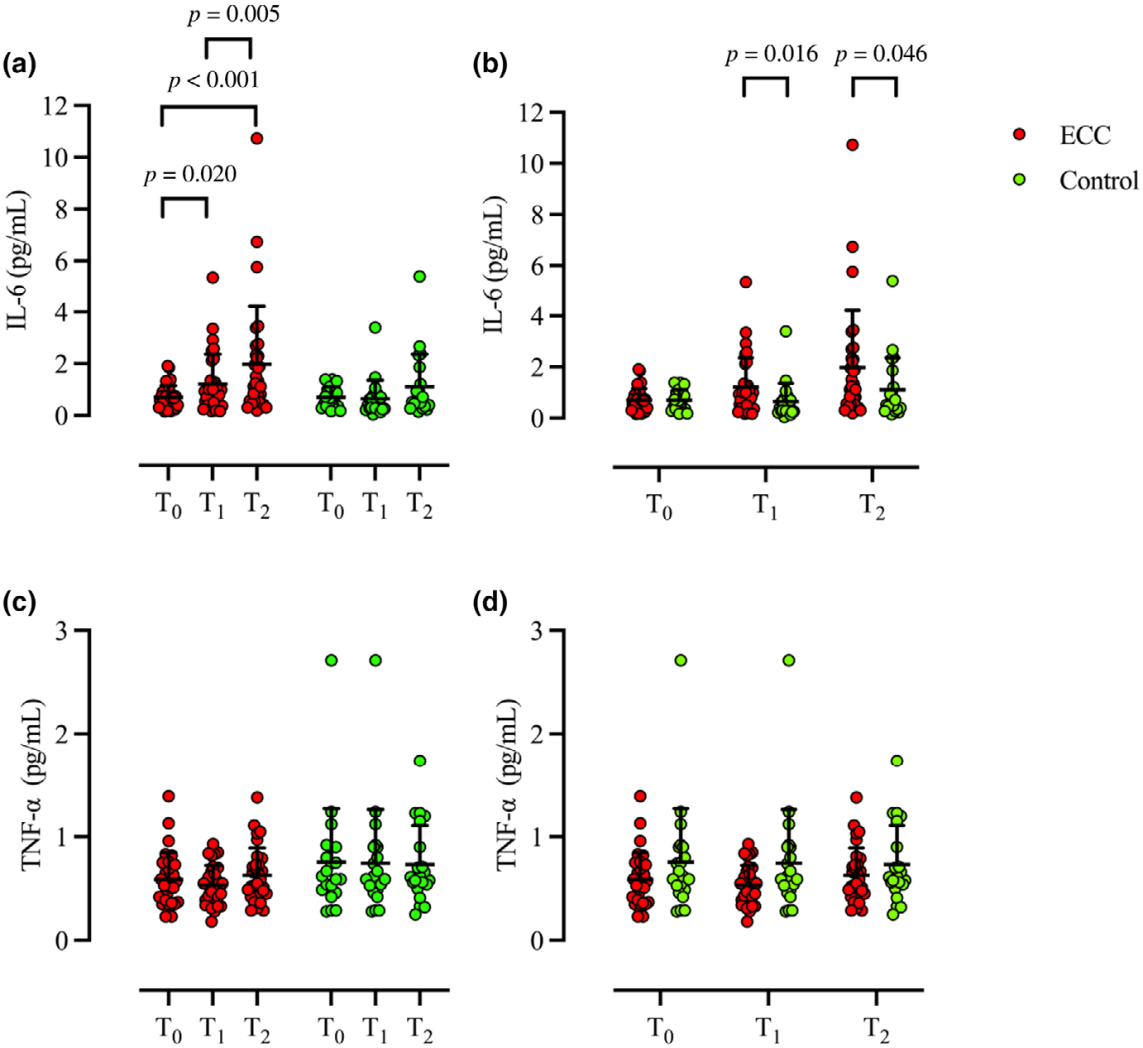
Individual (circle) and mean ± SD (black) concentrations of IL‐6 (a and b) and TNF‐α (c and d) comparison at timepoints T_0_, T_1_, and T_2_ within (a and c) and between (b and d) ECC (red) and Control (green). Wilcoxon signed‐rank test [within (a and c)] and Mann–Whitney *U*‐test [between (b and d)]; ECC, *n* = 31; Control, *n* = 21. ECC, eccentric exercise; IL‐6, Interleukin‐6; TNF‐α, tumor necrosis factor alpha.

Significant differences were observed within ECC in concentrations of IL‐6 (*p* < 0.001). Specifically, levels were significantly elevated from T_0_ (0.70 ± 0.45 μg/L) to T_1_ (1.21 ± 1.45 μg/L, *p* = 0.020, *r* = 0.292) and to T_2_ (2.0 ± 2.23 μg/L, *p* < 0.001, *r* = 0.508), and from T_1_ to T_2_ (*p* = 0.005, *r* = 0.355). No difference was observed in concentrations of IL‐6 within control (T_0_ 0.70 ± 0.46 μg/L; T_1_ 0.64 ± 0.72 μg/L; T_2_ 1.11 ± 1.24 μg/L, *p* = 0.066). There was no difference between ECC and control at T_0_ (*p* = 0.933); however, IL‐6 levels were higher in ECC at T_1_ (*p* = 0.016, *r* = 0.332) and T_2_ in ECC compared to control (*p* = 0.046, *r* = 0.276) (Figure 2). No difference in concentrations of TNF‐α was observed within ECC (T_0_ 0.59 ± 0.26 μg/L, T_1_ 0.53 ± 0.19 μg/L; T_2_ 0.63 ± 0.26 μg/L, *p* = 0.206) or control (T_0_ 0.75 ± 0.51 μg/L, T_1_ 0.74 ± 0.52 μg/L; T_2_ 0.73 ± 0.37 μg/L *p* = 0.939). There was no difference between ECC and control at T_0_ (*p* = 0.236), T_1_ (*p* = 0.099), or T_2_ (*p* = 0.386) (Figure 2). IL‐1β was not detectable and remained below the assay range of the tested kit (0.1–8 pg/mL).

**FIGURE 3:**
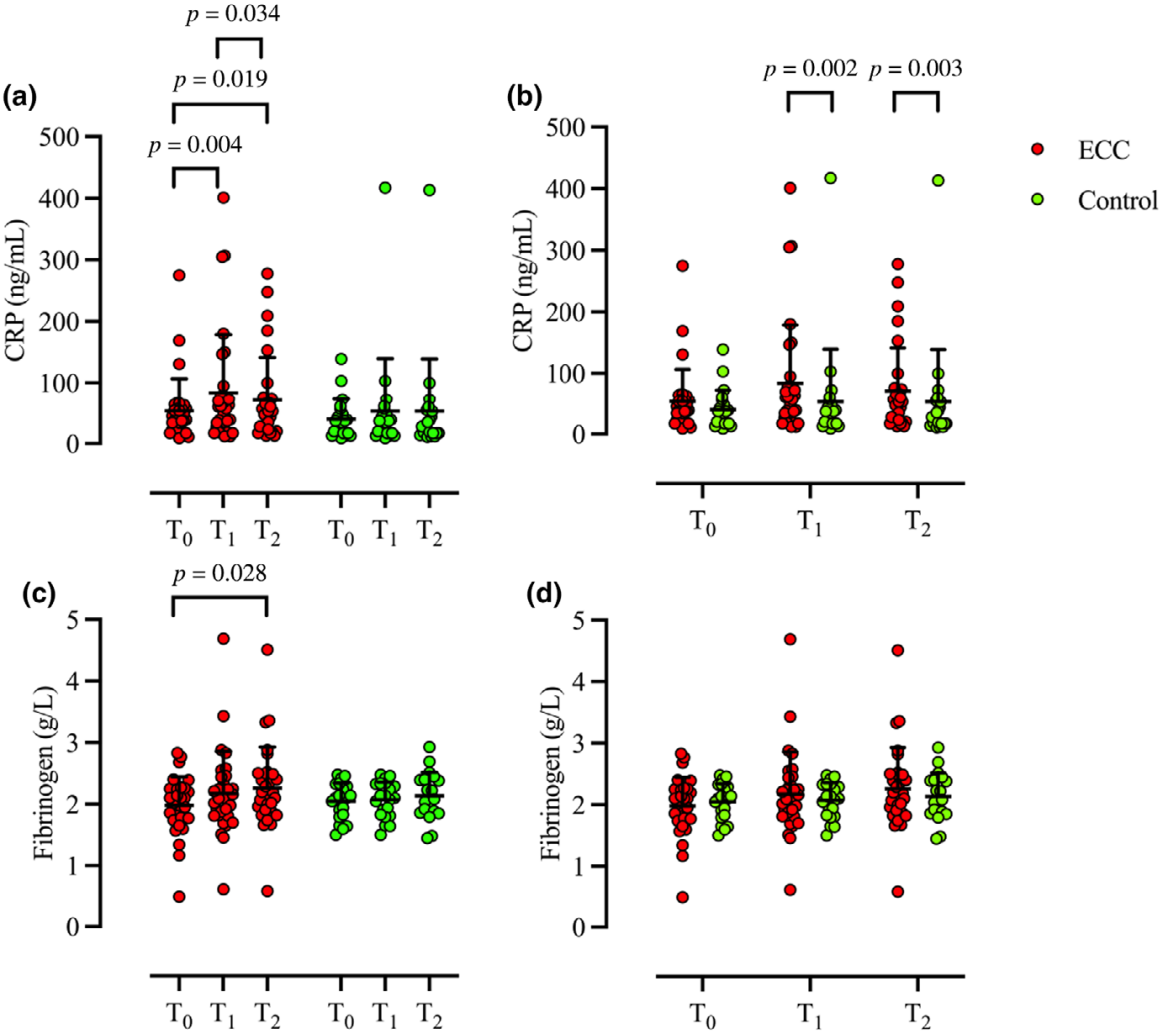
Individual (circle) and mean ± SD (black) concentrations of CRP (a and b) and fibrinogen (c and d) comparison at timepoints T_0_, T_1_, and T_2_ within (a and c) and between (b and d) ECC (red) and control (green). Wilcoxon signed‐rank test [within (a and c)] and Mann–Whitney *U*‐test [between (b and d)]; ECC, *n* = 31; Control, *n* = 21. CRP, c‐reactive protein; ECC, eccentric exercise.

Significant differences in CRP levels were observed within ECC (*p* = 0.001), with elevated levels from T_0_ (62 ± 54 ng/mL) to T_1_ (101 ± 92 ng/mL, *p* = 0.004, *r* = 0.369) and T_2_ (87 ± 70 ng/mL, *p* = 0.019, *r* = 0.285), but lower concentrations at T_2_ compared to T_1_ (*p* = 0.034, *r* = 0.266). No difference in CRP levels were observed between any time points within Control (T_0_ 49 ± 31 ng/mL; T_1_ 52 ± 86 ng/mL; T_2_ 53 ± 85 ng/mL, *p* = 0.291). There was no difference between ECC and Control at T_0_ (*p* = 0.101), but higher concentrations were noted in ECC at T_1_ (*p* = 0.002, *r* = 0.439) and T_2_ (*p* = 0.003, *r* = 0.416) (Figure 3). Significant differences were noted within ECC in concentrations of fibrinogen (*p* = 0.034), with higher levels at T_2_ (2.26 ± 0.66 g/L) than T_0_ (1.98 ± 0.46 g/L, *p* = 0.028, *r* = 0.278), but no difference between T_0_ and T_1_ (2.17 ± 0.68 g/L, *p* = 0.131) nor between T_1_ and T_2_ (*p* = 0.327). There was no difference in concentrations of fibrinogen within Control (T_0_ 2.0 ± 0.29 g/L; T_1_ 2.1 ± 0.27 g/L; T_2_ 2.2 ± 0.38 g/L, *p* = 0.995). There was no difference between ECC and Control at T_0_ (*p* = 0.608), T_1_ (*p* = 0.773), or T_2_ (*p* = 0.621) (Figure 3).

### 3.3 Biomarkers – VGE_visible_ and VGE_no‐visible_

**FIGURE 4:**
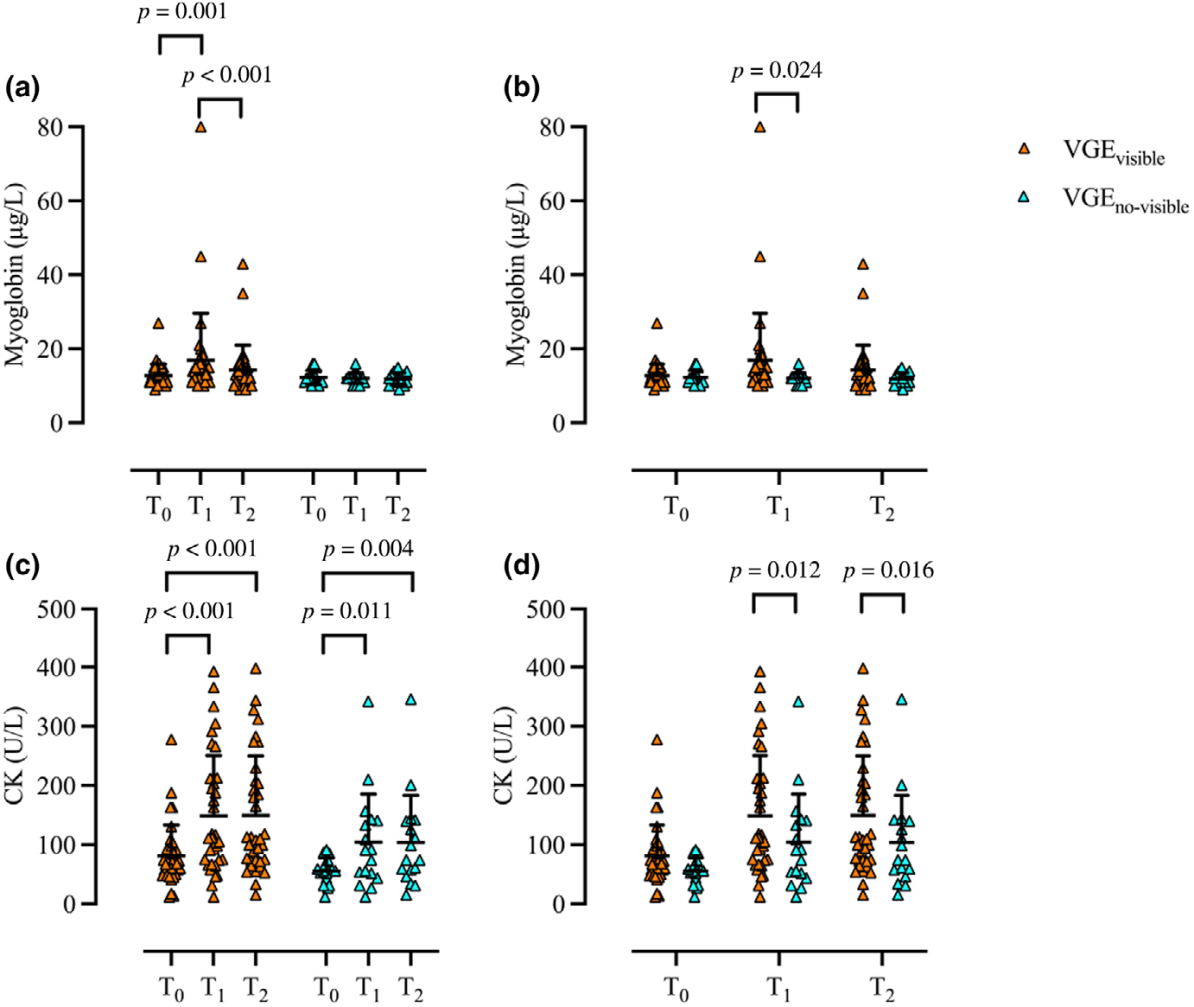
Individual (rectangle) and mean ± SD (black) concentrations of myoglobin (a and b) and CK (c and d) comparison at timepoints T_0_, T_1_, and T_2_ within (a and c) and between (b and d) VGE_visible_ (orange) and VGE_no‐visible_ (blue). Wilcoxon signed‐rank test [within (a and c)] and Mann–Whitney *U*‐test [between (b and d)]; VGE_visible_, *n* = 35, VGE_no‐visible_, *n* = 17. CK, creatine kinase.

Significant differences in myoglobin levels were found within VGE_visible_ (*p* < 0.001). Specifically, myoglobin increased from T_0_ (12.7 ± 3.1 μg/L) to T_1_ (16.9 ± 12.6 μg/L, *p* = 0.001, *r* = 0.372) and decreased from T_1_ to T_2_ (14.3 ± 6.7 μg/L, *p* < 0.001, *r* = 0.428), while no difference was observed between T_0_ and T_2_ (*p* = 0.128). Within VGE_no‐visible_, no difference in myoglobin were observed between any time points (T_0_ 12.2 ± 1.8 μg/L; T_1_ 13.1 ± 1.4 μg/L, T_2_ 10.8 ± 1.7 μg/L, *p* = 0.366). There was no difference in myoglobin levels at T_0_ (*p* = 0.805) or T_2_ (*p* = 0.175) comparing VGE_visible_ and VGE_no‐visible_, however, myoglobin was significantly higher at T_1_ in VGE_visible_ compared to VGE_no‐visible_ (*p* = 0.024) (Figure 4). CK levels were significantly increased within both VGE_visible_ (*p* < 0.001) and VGE_no‐visible_ (*p* = 0.002). Specifically, levels were increased in both groups from T_0_ (VGE_visible_, 81 ± 52 U/L; VGE_no‐visible_, 75 ± 21 U/L) compared to T_1_ (VGE_visible_, 149 ± 102 U/L, *p* < 0.001, *r* = 0.466; VGE_no‐visible_, 89 ± 56 U/L, *p* = 0.011, *r* = 0.435) and T_2_ (VGE_visible_, 150 ± 100 U/L, *p* < 0.001, *r* = 0.470; VGE_no‐visible_, 88 ± 51 U/L, *p* = 0.004, *r* = 0.497), while no differences were observed between T_1_ and T_2_ (VGE_visible_, *p* = 0.995; VGE_no‐visible_, *p* = 0.669). There was no difference in concentrations of CK at T_0_ (*p* = 0.180); however, CK levels were higher at T_1_ in VGE_visible_ (*p* = 0.012, *r* = 0.332) and T_2_ (*p* = 0.016, *r* = 0.334) (Figure 4).

**FIGURE 5:**
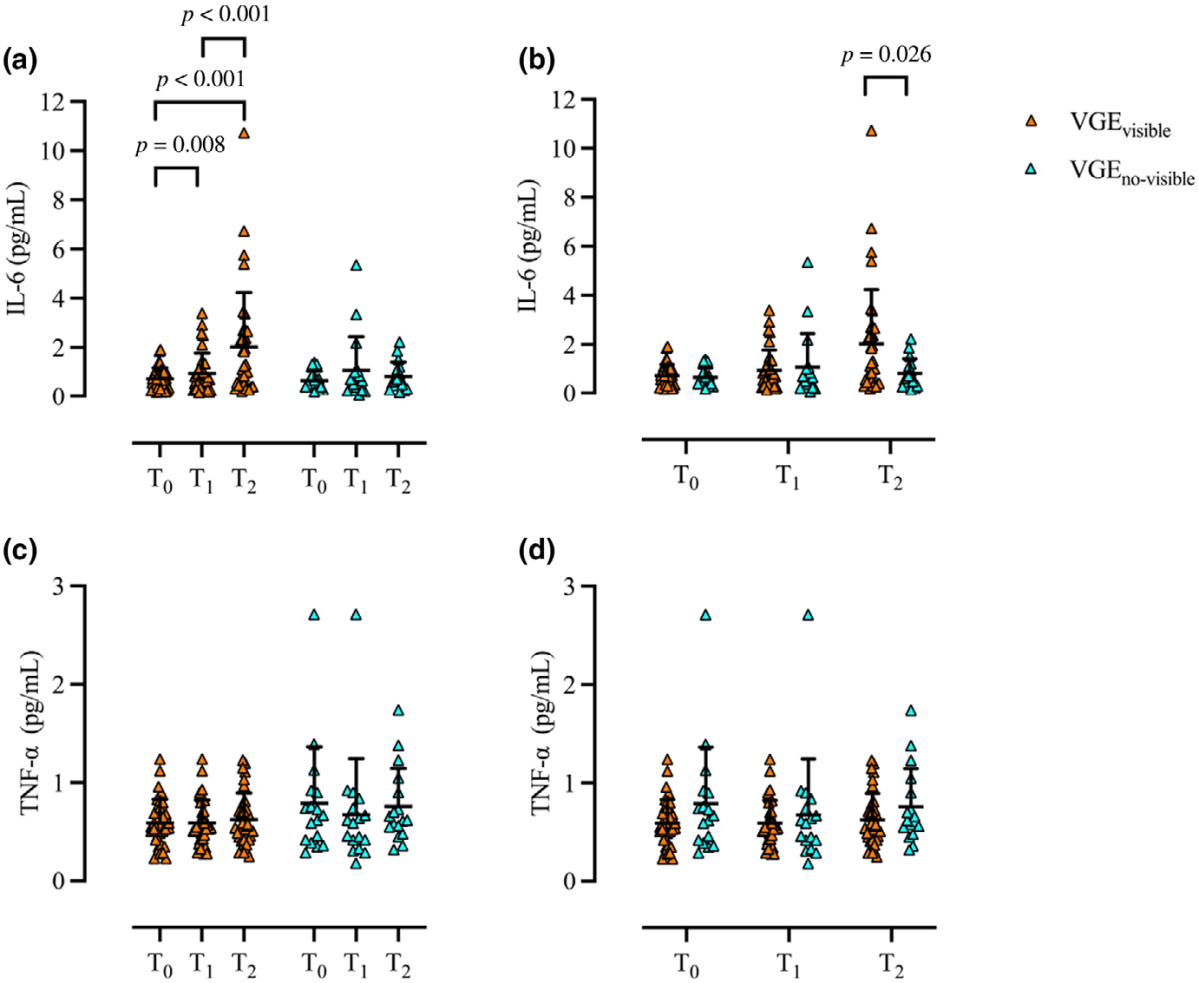
Individual (rectangle) and mean ± SD (black) concentrations of IL‐6 (a and b) and TNF‐α (c and d) comparison at timepoints T_0_, T_1_, and T_2_ within (a and c) and between (b and d) VGE_visible_ (orange) and VGE_no‐visible_ (blue). Wilcoxon signed‐rank test [within (a and c)] and Mann–Whitney *U*‐test [between (b and d)]; VGE_visible_, *n* = 35, VGE_no‐visible_, *n* = 17. IL‐6, Interleukin‐6, TNF‐α, Tumor necrosis factor alpha.

Within VGE_visible_, significant differences in IL‐6 were noted (*p* < 0.001), with elevated levels from T_0_ (0.59 ± 0.54 μg/L) to T_1_ (0.89 ± 0.79 μg/L, *p* = 0.008, *r* = 0.554) and T_2_, (1.97 ± 2.23 μg/L, *p* < 0.001, *r* = 0.497), and between T_1_ and T_2_ (*p* < 0.001, *r* = 0.529). There was no difference in IL‐6 levels within VGE_no‐visible_ (T_0_ 0.65 ± 0.38 μg/L; T_1_ 1.07 ± 1.38 μg/L; T_2_ 0.81 ± 0.59 μg/L, *p* = 0.662). There was no difference in IL‐6 levels between VGE_visible_ and VGE_no‐visible_ at T_0_ (*p* = 0.725) or T_1_ (*p* = 0.605), but levels were higher in VGE_visible_ at T_2_ (*p* = 0.026, *r* = 0.309) (Figure 5). There was no difference in TNF‐α levels within VGE_visible_ (*p* = 0.670) or VGE_no‐visible_ (*p* = 0.178). There was no difference in concentrations of TNF‐a at T_0_ (*p* = 0.254), T_1_ (*p* = 0.992), or T_2_ (*p* = 0.238) (Figure 5).

**FIGURE 6:**
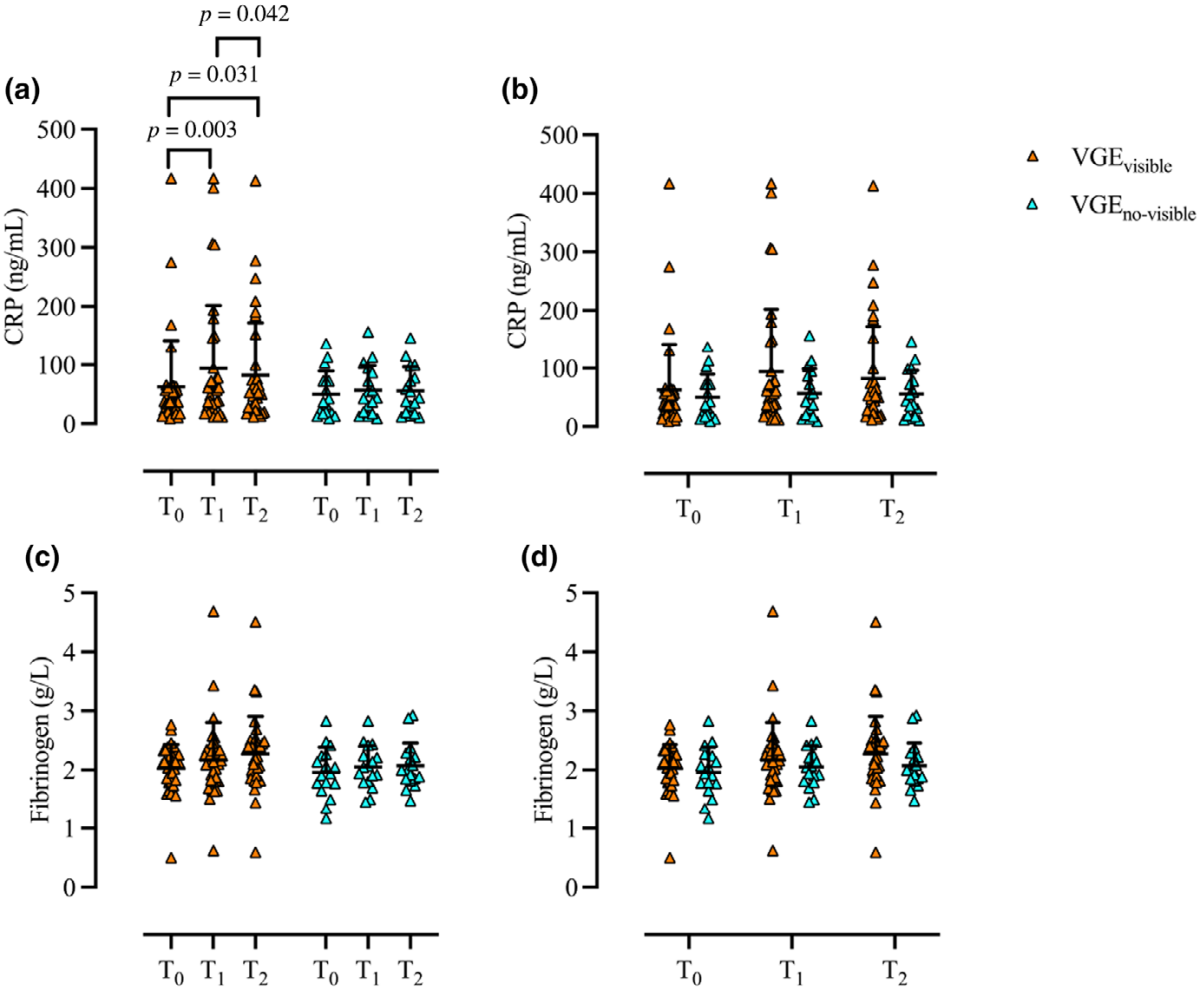
Individual (rectangle and rhomb) and mean ± SD (black) concentrations of CRP (a and b) and fibrinogen (c and d) comparison at timepoints T_0_, T_1_, and T_2_ within (a and c) and between (b and d) VGE_visible_ (orange) and VGE_no‐visible_ (blue). Wilcoxon signed‐rank test [within (a and c)] and Mann–Whitney *U*‐test [between (b and d)]; VGE_visible_, *n* = 35, VGE_no‐visible_, *n* = 17. CRP, C‐reactive protein.

Significant differences were observed in CRP levels within VGE_visible_ (*p* = 0.651). Specifically, CRP levels were increased from T_0_ (62 ± 79 ng/mL) at T_1_ (94 ± 107 ng/mL, *p* = 0.003, *r* = 0.356) and T_2_ (83 ± 90 ng/mL, *p* = 0.031, *r* = 0.257), while reduced from T_1_ to T_2_ (*p* = 0.042, *r* = 0.242). There was no difference in CRP levels within VGE_no‐visible_ (p = 0.651). There was no difference in CRP levels between the groups at T_0_ (*p* = 0.884), T_1_ (*p* = 0.441), or T_2_ (*p* = 0.526) (Figure 6). No difference was noted in fibrinogen levels within VGE_visible_ (*p* = 0.124) or VGE_no‐visible_ (*p* = 0.504). There was no difference in fibrinogen levels between the groups at T_0_ (*p* = 0.413), or T_1_ (*p* = 0.482) or T_2_ (*p* = 0.105) (Figure 6).

### 3.4 Correlations

There was a significant correlation between T_1_ myoglobin levels and VGE_max_ (*p* = 0.004, *r*_*s*_ = 0.454) and KISS (*p* = 0.002, *r*_*s*_ = 0.505). In addition, the percentage change in myoglobin from T_0_ to T_1_ was significantly correlated with VGE_max_ (*p* < 0.001, *r*_*s*_ = 0.450) and KISS (*p* < 0.001, *r*_*s*_ = 0.506). There was a significant correlation between changes in T_1_‐T_2_ IL‐6 levels and VGE_max_ (*p* = 0.033; *r*_*s*_ = 0.396), but not KISS (*p* = 0.155). There was no correlation between VGE_max_ or KISS and T_1_ concentrations of CK, IL‐6, TNF‐α, CRP, or fibrinogen. There was no correlation between VGE_max_ or KISS and absolute or percentage changes of CK, TNF‐α, CRP, or fibrinogen between T_1_ and T_2_.

## 4 DISCUSSION

The aim of this study was to investigate the effect of eccentric exercise on muscle damage and inflammation and explore their possible role on high‐altitude‐induced VGE. Present results demonstrate that eccentric EIMD and inflammation aggravates decompression strain during a continuous 90‐min exposure at 24,000 ft. Furthermore, our findings signify that VGE load further exacerbate the degree of systemic inflammation, with a positive correlation being noted between VGE_max_ and IL‐6, a finding that was absent in the control condition where a lower decompression strain was noted.

The exposures preceded by eccentric exercise were associated with a higher decompression strain compared with control. Specifically, both maximal VGE scores and KISS were higher in the ECC compared to Control. The ECC protocol and VGE_visbile_ group were associated with a state of EIMD as noted by the elevation in CK and myoglobin levels from T_0_ to T_1_ (Figure 1 and Figure 4). Notably, concentrations of CK and myoglobin were higher at T_1_ compared to Control and VGE_no‐visible_, respectively (Figure 1 and Figure 4). In addition, the relative change in myoglobin from T_0_ to T_1_ and the absolute value at T_1_ were correlated to both VGE_max_ and KISS. Although concentrations of CRP and IL‐6 increased within ECC and VGE_visible_, levels were only higher compared to Control values at T_1_ in ECC. This implies that ECC was also associated with inflammation; however, no correlations were found between levels of these markers prior to the hypobaric exposure (T_1_) and VGE_max_ or KISS. Taken together, our results suggests that EIMD was associated with high‐altitude‐induced VGE formation. The question that then arises is why eccentric EIMD is associated with decompression strain?

It is generally accepted that supersaturation alone is not sufficient to create the VGE observed during decompression, as de novo bubble formation requires pronounced levels of supersaturation (>100 ATA) (Jones et al., 1999). Instead, the main hypothesis is that saturated gas enters pre‐existing gas pockets (i.e., micronuclei) upon decompression, which then enters the circulation and grow until they become visible VGE. Thus, the combined findings of this and previous studies (Gottschalk et al., 2023; Gottschalk, Gennser, Günther, et al., 2024) suggests that eccentric contractions, resulting in EIMD, influence creation and/or growth of micronuclei, and different mechanisms may be involved. Firstly, eccentric contractions can result in microscopic tears in connective tissue of mucle fibers and tendons. These structural changes might lead to more sites (e.g., crevices) for micronuclei to adhere to (Tikuisis, 1986). Furthermore, damage to the endothelial cells in muscles could lead to increased release of microparticles (MPs), which may, in turn, contribute to a higher occurrence of bubbles (Thom et al., 2013). However, no measuments of MPs were taken in the current study, but the possible relationship between MPs and VGE formation warrants further investigation. In addition, inflammation and local microvascular hyperpermeability due to EIMD could enable the micronuclei to more easily transverse into the vascular system (Hotta et al., 2018; Muller, 2011; Vestweber, 2015).

Alternatively, higher VGE scores after the ECC could result from increased nucleation caused by localized negative pressures in tissues (muscle and tendon attachments), induced by mechanical stress of eccentric contrations. Nucleation occurs in biological systems even under normal atmospheric pressure, as seen in synovial joints when stretched beyond a certain point, wherein a vapor‐filled bubble is generated (Kawchuk et al., 2015). Furthermore, signals indicative of microbubbles have been detected following exercise using dual‐frequency ultrasound imaging (Wilbur et al., 2010), suggesting that nucleation can also rise from muscle and tendon movement, not just in joints (Harvey, 1945; Unsworth et al., 1971; Whitaker et al., 1945). Thus, myoglobin levels could be correlated to increased VGE formation not because of the muscle damage per se, but rather due to the eccentric exercise that has induced it. Thus, the eccentric exercise may cause both muscle damage and cavitation phenomena. The former will lead to an increased blood concentration of myoglobin while the latter will increase the prevalence of VGE. It is reasonable to assume that the elevation in myoglobin reflects the exertion during the eccentric exercise and, consequently, the potential for cavitation.

Yet another mechanism could involve elevated CO_2_ levels which has been suggested to facilitate bubble formation (Daubresse et al., 2024; Harris et al., 1945; Jauchem, 1988). However, it is unlikely that systemic CO_2_ levels would be elevated 24 h after the exercise intervention (Williams & Horvath, 1995). Additionally, CO_2_ production is typically greater during concentric rather than eccentric exercise, as the former requires more energy and involves higher metabolic activity (Herzog, 2018; Perrey et al., 2001). This is particularly evident in aerobic exercise (such as running and cycling), which have not shown an increase in VGE formation following decompression (Blatteau et al., 2005; Gennser et al., 2012). Instead, it is possible that the EIMD may raise lactate levels which in turn elevate CO_2_ as the body buffers acidity (Asp et al., 1998). This, combined with impaired vascular function (Caldwell et al., 2016; Kano et al., 2005), could create conditions favorable for VGE formation due to localized supersaturation. Also, reduced nitric oxide (NO) bioavailability may influence VGE formation. NO‐donors have been shown to reduce VGE incidence post‐decompression (Dujić et al., 2006; Møllerløkken et al., 2006), while NO‐synthase inhibition increases bubble formation (Wisløff et al., 2003). Eccentric EIMD impairs endothelial function (Stacy et al., 2013), likely due to elevated levels of reactive oxygen species, which in turn reduce NO availability (Förstermann & Sessa, 2012). Moreover, CRP, which was significantly higher in the ECC than Control at T_1_, has been observed to reduce the expression of endothelial‐derived NOS (Schwartz et al., 2007; Venugopal et al., 2002).

Lastly, a potential mechanism to be considered here is the effect of ECC on the micronuclei surface tension. Surfactants are known for their ability to reduce surface tension between two substances, such as between liquid and gas. Proteins share this surfactant‐like property due to their combined hydrophilic and hydrophobic regions. Myoglobin, for instance, has been proposed to affect molecular orientation at membrane interfaces (Ahmed et al., 2023; Tofani et al., 2004). Thus, it is tempting to speculate that myoglobin could coat existing micronuclei, prolonging their lifespan and allowing for increased nitrogen influx during decompression. This would explain, at least in part, why myoglobin levels pre‐altitude exposure were higher in VGE_visible_, as well as the correlation found with VGE_max_ and KISS. It is noteworthy that myoglobin levels were reduced from T_1_ to T_2_ in ECC and VGE_visbile_ but not Control or VGE_no‐visible_. It is possible that the surfactant‐like properties of myoglobin caused it to adhere to the VGE, leading to its reduction from T_1_ levels. An alternative explanation may be that the release of myoglobin from damaged tissues is reduced due to the vasoconstrictive effect of hyperoxia (Brugniaux et al., 2018). This may also help explain why CK levels were not reduced at T_2_, as CK, due to its size, cannot as easily enter the vascular system and is primarily taken up by the lymphatic system (Lindena et al., 1979).

Concentrations of IL‐6 were increased from T_1_ to T_2_ in both the ECC and VGE_visible_ groups, while no differences were observed in Control or VGE_no‐visible_. These findings align with previous studies conducted following hyper‐ and hypobaric decompression (Connolly et al., 2023; Rocco et al., 2021; Žarak et al., 2021). While neither Rocco et al. nor Zarak et al. performed measurements of VGE after their respective diving interventions, consistent with Conolly et al., we noted a relationship between VGE and IL‐6 (Connolly et al., 2023). IL‐6 is a pro‐inflammatory cytokine produced by various cell types, including endothelial cells, in response to mechanical stimuli (Matsuda & Kishimoto, 1998). Therefore, elevations in IL‐6 following decompression are likely due to mechanical stress exerted by circulating VGE on endothelial cells, consequently initiating an inflammatory response. Contrastingly, no differences were noted in TNF‐α before and after the hypobaric exposure. Our findings are in agreement with those of Rocco et al. (2021) which also failed to report any changes in the circulating concentration of TNF‐α following a 2‐h open water dive. These lack of changes probably relate to the short half‐life (few minutes) of this proinflammatory cytokine (Held et al., 2019); thus, since blood samples were drawn immediately following decompression, the time taken for recompression (~5 min) might have been too long to detect any possible changes.

Contrary to prior observations following hypo‐ and hyperbaric decompression (Connolly et al., 2023; Žarak et al., 2021), we observed a slight reduction in T_1_‐T_2_ CRP levels in ECC and VGE_visible_ (Figures 3 and 6). However, it should be noted that these differences may stem from distinct differences in both the experimental designs [e.g., two consecutive exposures at 25,000 ft. (Connolly et al., 2023); 30‐min dives at 20–30 m (Žarak et al., 2021)] as well as the timing of the blood collections [e.g., 8‐h and 24‐h post the hypobaric exposure (Connolly et al., 2023)]. Nonetheless, our findings are in part agreement with those noted after hyperbaric oxygen (HBO) therapy (De Wolde et al., 2021). Although CRP reductions were observed following multiple HBO sessions (De Wolde et al., 2021), it is possible that the present reductions may result from oxygen breathing, acutely mitigating the underlying inflammatory EIMD. Moreover, similar to findings following diving, we observed no changes in fibrinogen levels after decompression neither in subjects with visible VGE or no observable VGE (Kiboub et al., 2018).

In summary, this study suggests that eccentric EIMD and inflammation are associated with a higher decompression strain during a 90‐min continuous exposure at 24,000 ft. Furthermore, VGE load seems to further exacerbate the degree of systemic inflammation in a dose‐dependent manner.

## AUTHOR CONTRIBUTIONS

F.G., M.Ge., O.E., and A.E. contributed to the conception and design of the research. F.G., M.Ge., M.Gü., O.E., and A.E. conducted experiments. F.G. and A.E. performed data analysis. F.G., M.Ge, and A.E interpreted the results. F.G. drafted the manuscript. F.G., M.Ge., and A.E. edited and revised the manuscript. All authors approved the final version of the manuscript and agree to be accountable for all aspects of the work in ensuring that questions related to the accuracy or integrity of any part of the work are appropriately investigated and resolved. All persons designated as authors qualify for authorship, and all those who qualify for authorship are listed.

## CONFLICT OF INTEREST STATEMENT

The authors report there are no competing interests to declare.

## Data Availability

Data supporting the study findings may be requested from the corresponding author (F.G) but are not publicly available since they contain information that could compromise the privacy of the research participants.
